# A New Behavioral Test and Associated Genetic Tools Highlight the Function of Ventral Abdominal Muscles in Adult *Drosophila*

**DOI:** 10.3389/fncel.2017.00371

**Published:** 2017-11-21

**Authors:** Marine Pons, Claire Soulard, Laurent Soustelle, Marie-Laure Parmentier, Yves Grau, Sophie Layalle

**Affiliations:** Institut de Génomique Fonctionnelle, CNRS, INSERM, Université de Montpellier, Montpellier, France

**Keywords:** neuromuscular junction, motor neuron, muscle, behavior, *Drosophila*

## Abstract

The function of the nervous system in complex animals is reflected by the achievement of specific behaviors. For years in *Drosophila*, both simple and complex behaviors have been studied and their genetic bases have emerged. The neuromuscular junction is maybe one of the prototypal simplest examples. A motor neuron establishes synaptic connections on its muscle cell target and elicits behavior: the muscle contraction. Different muscles in adult fly are related to specific behaviors. For example, the thoracic muscles are associated with flight and the leg muscles are associated with locomotion. However, specific tools are still lacking for the study of cellular physiology in distinct motor neuron subpopulations. Here we decided to use the abdominal muscles and in particular the ventral abdominal muscles (VAMs) in adult *Drosophila* as new model to link a precise behavior to specific motor neurons. Hence, we developed a new behavioral test based on the folding movement of the adult abdomen. Further, we performed a genetic screen and identify two specific Gal4 lines with restricted expression patterns to the adult motor neurons innervating the VAMs or their precursor cells. Using these genetic tools, we showed that the lack of the VAMs or the loss of the synaptic transmission in their innervating motor neurons lead to a significant impairment of the abdomen folding behavior. Altogether, our results allow establishing a direct link between specific motor neurons and muscles for the realization of particular behavior: the folding behavior of the abdomen in *Drosophila*.

## Introduction

The muscle system accounts for almost half of the body mass in animals. Vertebrate muscles are usually subdivided into three types related to their different functional properties: the skeletal, cardiac, and the smooth muscles. In *Drosophila* the counterparts of these muscle categories are also found and are known as somatic (or body wall muscles), heart, and visceral muscles. Even if the final shapes and functions of muscles differ between vertebrates and *Drosophila*, the myogenesis processes share common features (Taylor, 2006). The development and the formation of the different muscle types have been extensively studied for years in *Drosophila* (for review Roy and VijayRaghavan, 1999; Tixier et al., 2010; Dobi et al., 2015; Bothe and Baylies, 2016). *Drosophila* is a holometabolous insect with two motile lives: the larva is able to crawl and the adult can walk and fly. During metamorphosis the muscle system needs to be remodeled to completely switch its shape, implying drastic changes. Almost all the larval muscles degenerate and adult muscles develop *de novo* to construct the final stereotyped pattern of the adult fly. Among the different muscles forming the adult fly, the thoracic muscles have been the most studied to understand the regulation and the function of many proteins involved in muscle activity (for example the myofibril formation Vigoreaux, 2001; Schnorrer and Dickson, 2004). These thoracic muscles include the IFMs (Indirect Flight Muscles), the DFMs (Direct Flight Muscles) and the jump muscle (Miller, 1950; Crossley, 1978; Lawrence, 1982; Fernandes et al., 1991). The leg muscles, due to their small size are more difficult to dissect and to observe and until recently, have not been routinely employed for physiological or developmental studies (Enriquez et al., 2015; Soler et al., 2016; Syed et al., 2016).

Interestingly, various behavioral tests are associated with the different adult muscle functions. For example, the flight of the fly can be studied to understand the IFMs function (Drummond et al., 1991; Cripps et al., 1994). The initial jumping behavior occurring pre-flight during takeoff can also be analyzed and be used as a read-out of the function of the jump muscle (Elliott et al., 2007). Finally, the adult fly locomotion can also be monitored as a read-out of leg muscle activity, using the negative geotaxis behavioral test that measures the celerity of the fly walk (Benzer, 1967; Whitworth et al., 2005). More recently, the gait in a freely walking fly has also be analyzed using a footprint tracking test (Gargano et al., 2005; Mendes et al., 2013). In contrast to the leg or the thoracic muscles, very little is known about the function of the abdominal muscles, although these muscles cover the inner surface (ventrally, laterally and dorsally) of the adult abdomen. Although they are probably involved in abdomen movements, there is no report of the study of their function and, in particular, no associated behavioral test read-out to study their function. Miller in 1950 and Currie in 1991 have described these abdominal muscles anatomically (Miller, 1950; Currie and Bate, 1991). They have classified the abdominal muscles into three groups depending on their spatial location in the abdomen: dorsal, lateral and ventral. The muscle pattern is found repeated from segments A2–A7 and it is slightly different in segments A1 and A8. Dorsal and ventral muscles are organized in longitudinal fibers, whereas lateral muscles are arranged in parallel transverse fibers perpendicular to the ventral midline. Each hemisegment of the ventral muscles contains a group of 5–8 fibers orientated longitudinally to the ventral midline. The development and the histology of these muscles have raised interest (Currie and Bate, 1991), but so far the function of the abdominal muscles has not been addressed in adult fly.

Because among the abdominal muscles, the ventral muscles appear the largest and the most accessible with which to begin studying the function, we first analyzed the neuromuscular system of the adult ventral abdominal muscles (VAMs). We then searched for a behavioral read-out associated with the function of VAMs in the adult fly. On one hand, we designed a new behavioral test aimed at analyzing the folding movements of the adult abdomen. On the other hand, we characterized and used new genetic tools to be able to demonstrate the implication of the VAMs in this behavioral output. To that extent, by screening the Janelia Flight Light Gal4 lines (Pfeiffer et al., 2008), we identified two Gal4 lines with specific patterns: one targets the adult ventral muscle precursor cells and the other is specifically expressed in the motor neurons innervating the VAMs. Using these tools that specifically alter the ventral abdominal muscle functions, we here show that the activation of the VAMs is necessary to ensure the folding movements of the abdomen.

## Materials and methods

The following strains were used: Janelia Gal4 lines 24G08 Gal4 (Bloomington stock 49316) and 31B08 Gal4 (Bloomington stock 49351), Tdc2 Gal4 (Bloomington stock 9313), VGlut Gal4 (Bloomington stock 26160), VGlut MiMIC RMCE line with EGFP reporter (and GFDTF tagged) (Nagarkar-Jaiswal et al., 2015) (Bloomington stock 59411), UAS mGFP (Bloomington stocks 35839), UAS reaper (Bloomington stock 5824), UAS TeTxLC (Bloomington stock 28997). All crosses were raised at 25°C. The white Canton S (w^CS^) strain (outcrossed to CS, from Jean-Maurice Dura, IGH Montpellier, France) was used as wild type control and was crossed with the different Gal4 lines for control experiments.

### Immunohistochemistry and image acquisition

Adult female abdomens were dissected in PBS, 1 mM EDTA and fixed in 4% formaldehyde then washed with PBS 0.3% Triton (PBT), blocked in 1% solution of BSA (PBT-BSA). Primary antibodies were incubated at 4°C overnight in PBT-BSA solution at different concentrations: mouse anti-Dlg 1/100 (Developmental Studies Hybridoma Bank), rabbit anti-GFP 1/1000 (Invitrogen), goat anti-HRP Cy3 1/500 (Jackson ImmunoResearch), phalloidin Alexa Fluor 647 1/1,000 (Molecular Probes). Secondary antibodies donkey anti-rabbit or -mouse Alexa Fluor 488 1/800 (Molecular Probes) was incubated 2 h at room temperature. Abdomens were mounted in glycerol 80%. For all experiments, images of abdominal segments from A3 to A5 were acquired using a confocal laser scanning microscope (Zeiss LSM780 or Leica SPE) and analyzed using Fiji software (Schindelin et al., 2012).

### Adult abdomen folding behavior and video recording

Adult female flies aged of 6 days were used for behavioral experiments. At the same time of day (the afternoon), they were quickly immobilized on ice for few minutes before being transferred to a glass coverslip and pasted on the back, head right side, using melted myristic acid (Sigma) pieces. Once pasted, flies recovered during 2 h in a wet box. The camera (Sony HDR-CX240) lens was focused on 4 flies and the flies were video-recorded during 1-min twice at 4-min intervals. For each fly, the movie sequences were analyzed with Fiji and converted to kymograph using the Multi Kymograph plugin. The dark pigmentation on the tergites provided landmarks to identify the different abdominal segments. For each fly at the resting position, a line was drawn from the segment A2–A6 (yellow line in **Figures 3A,B**) and this line was used as the axis to carry out the kymograph. When the abdomen was not at the resting position, the abdomen was considered up or in other words in movement. A white peak on the kymograph visualized this movement. The amplitude of the movement was assessed using the A5 and A3 segments as landmarks. If the white peak was restricted to the A5 segment, the abdomen rises slightly and the movement was considered as small. In contrast, if the white peak spread over the A3 segment then the abdomen moves up with a larger range and the movement was considered as large. The number of white peaks was counted on each fly kymograph and characterized small and large abdomen movements performed by the fly during 1 min. The maximal abdomen folding were the curving movements during which the abdomen reached a position perpendicular to the resting position and the number of curving movement was directly counted on the movie. Kymograph could be converted into a black and white picture. All the black pixels depicted the abdomen at the resting position and the white pixels the abdomen in movement not aligned with the horizontal position. From that picture we could infer the percentage of activity of the fly abdomen corresponding to the percentage of all white pixels related to the total number of pixel.

## Results

### The adult ventral abdominal muscles and their neuromuscular junctions

**Figure 1:**
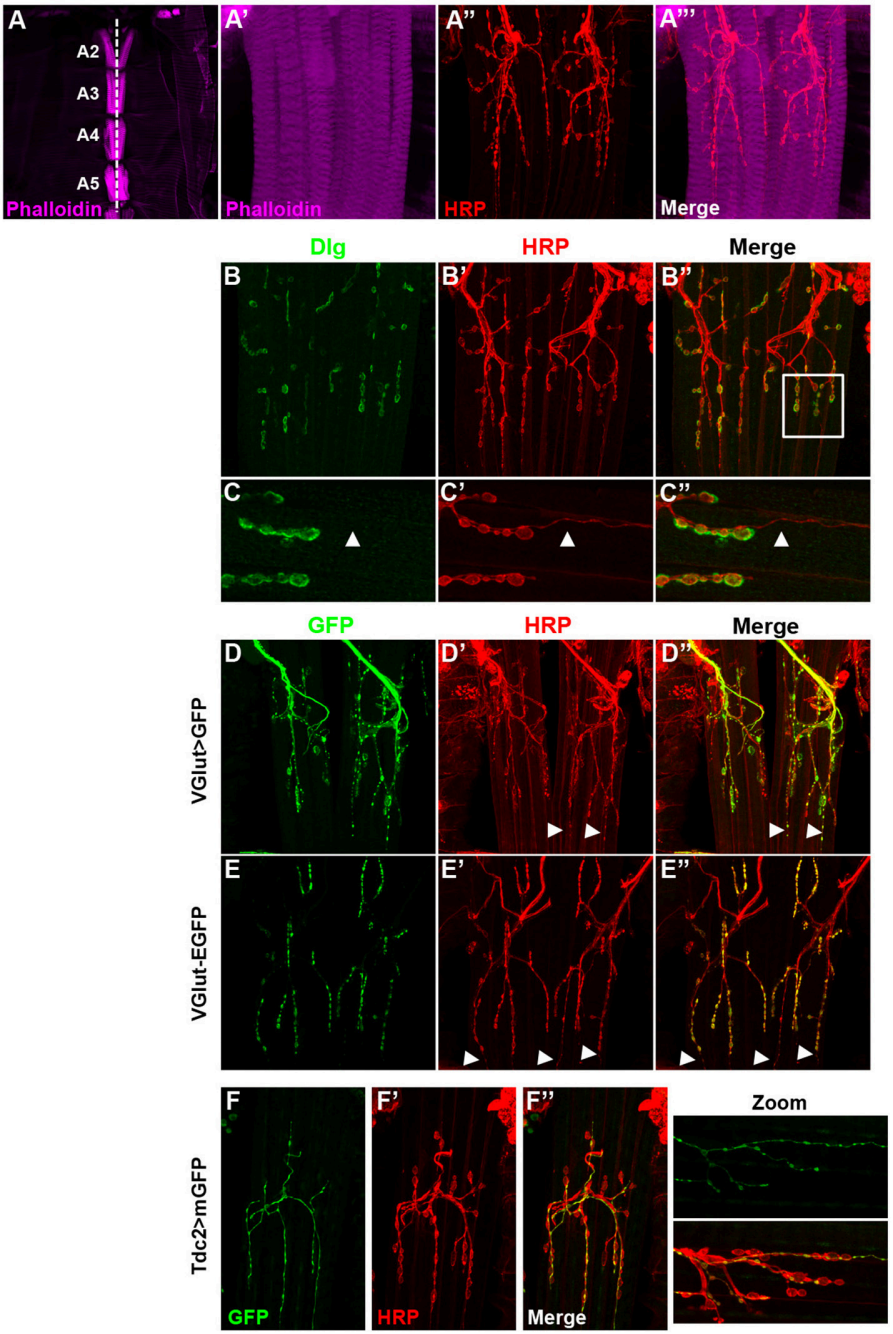
The ventral abdominal muscles (VAMs) and their innervation in adult *Drosophila*. **(A)** In wild type flies, the VAMs are organized in a stereotyped pattern on the abdomen as observed at low magnification (from A2 to A5, 10X objective). Muscles are labeled using a phalloidin staining (F-actin, in magenta). The white dotted line represents the ventral midline. **(A'–A”')** At higher magnification (63X objective) motor neurons (HRP staining in red) innervate each muscle fiber and synaptic boutons are visible. **(B–B”)** Discs large (Dlg, in green) is expressed at the post-synaptic level and surrounds the synaptic boutons (HRP labeled, in red). **(C–C”)** Zoom images of region outlined (white box in **B”**) show that Dlg **(C)** is not associated with the smaller boutons (**C'**, white arrowheads). In all presented images the anterior side is up. **(D–D”)** The VAMs are innervated by glutamatergic motors neurons. The VGlut Gal4 line drives the expression of the GFP in motor neurons and synaptic boutons of the VAMs. White arrowheads show the expression of GFP in small varicosities. **(E–E”)** The EGFP tagged VGlut construct labels all the synaptic boutons. The VGlut protein trap expression is very low in the smaller varicosities (white arrowheads). **(F–F”)** The VAMs receive also octopaminergic/tyraminergic innervation. **(F)** The Tdc2 Gal4 line allows the GFP expression exclusively in the smallest population of boutons (see merge in **F”**). This octopaminergic/tyraminergic innervation is closely associated to glutamatergic boutons as showed in zoomed images. For all conditions, motor neurons are stained with HRP (Horse Radish Peroxidase is a neuronal membrane marker, Jan and Jan, 1982), images of abdominal segments A3–A5 are represented.

The VAMs in adult fly are composed of several fibers lined up according to the antero-posterior axis on both sides of the ventral midline. At low magnification (Figure 1A), one set of ventral muscles is observable per hemisegment. The A2 segment shows a muscle-specific chevron pattern due to the presence of two clusters of chordotonal organs (Wheeler's organ) (Bodmer and Jan, 1987; Elliott et al., 2005; Eberl and Boekhoff-Falk, 2007). The VAMs are innervated by motor neurons (MNs) in a very stereotyped way that has been described in detail by two studies (Hebbar et al., 2006; Wagner et al., 2015 and Figures 1A'–A”'). Here, we further describe markers of these MNs, as well as the anatomy of their synaptic endings, the neuromuscular junctions (NMJs), in order to find and use appropriate Gal4 lines to study the function of these muscles. Previous studies did show that these adult NMJs are morphologically similar to the well-known larval ones (for review Prokop, 2006; Beramendi et al., 2007; Menon et al., 2013) and the commonly used synaptic markers remain expressed at these adult NMJs level (Beramendi et al., 2007; Wagner et al., 2015). As in larvae, at the postsynaptic part, the synaptic boutons make contact with muscles and are surrounded by infoldings of the muscle membrane named the subsynaptic reticulum (SSR) where the scaffolding protein Discs-large (Dlg) is found (Lahey et al., 1994; Budnik et al., 1996 and Figures 1B–B”). For branches specifically containing small synaptic boutons, the surrounding Dlg staining appears very faint, even absent (Figures 1C–C”, arrowheads in zoomed images), as it is also the case for specific boutons at the larval NMJ. As it is described in the larval neuromuscular system, the VAMs are innervated by glutamatergic synapses (Hebbar et al., 2006; Prokop, 2006). In accordance with this, we showed that the vesicular glutamate transporter, VGlut Gal4 driver (also known0 as OK371 Gal4) is broadly expressed in the MNs that target the VAMs (Figures 1D–D”). We also used the protein trap VGlut-EGFP to directly visualize the protein. This protein-trap line shows that the vesicular glutamate transporter is actually present at the synaptic terminals of the MNs innervating the VAMs (Figures 1E–E”). We also noticed the presence of small varicosities, HRP stained, that do not seem to be associated with SSR at the postsynaptic part since no Dlg staining is observed (Figures 1C–C” arrowheads in zoomed images). These boutons stay VGlut Gal4 positive even if the protein trap VGlut-GFP staining is quite null or very faint (Figures 1C–E” arrowheads). These characteristics suggest that these small varicosities could be octopaminergic/tyraminergic MNs innervations like type II boutons at the larval NMJ (Johansen et al., 1989; Rivlin et al., 2004). To confirm this hypothesis, we used a Tdc2 Gal4 line to target the expression of GFP in octopaminergetic/tyraminergic neurons (Cole et al., 2005; Koon et al., 2011) and found that all branches with small varicosities were actually GFP-positive (Figures 1F–F”). Hence these observations will enable the search for VAMs specific genetic tools and to target their function.

### Gal4 lines screen for the study of VAMs function

**Figure 2:**
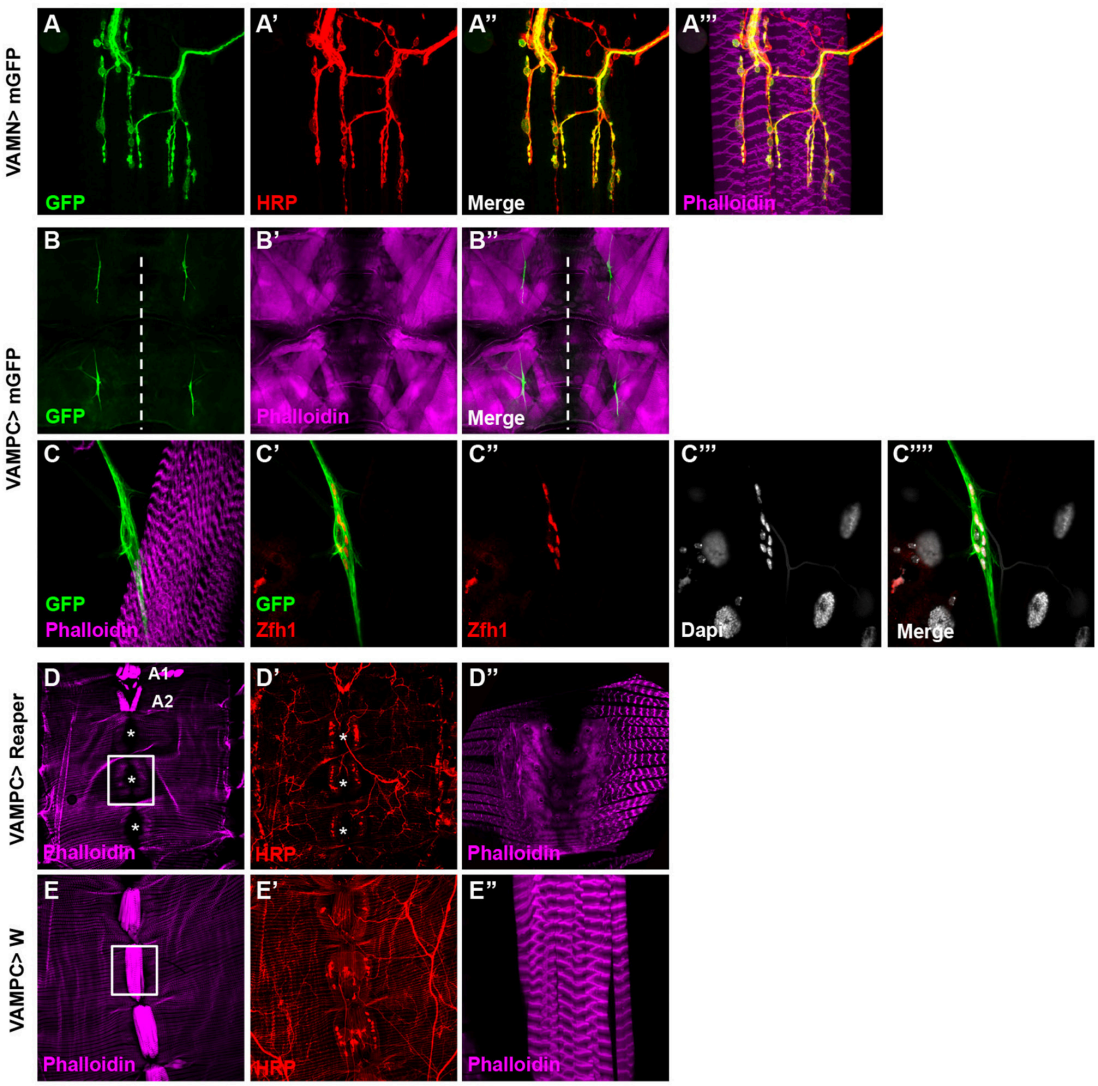
Identification of specific Janelia Gal4 lines related to the VAMs. **(A–A”')** The 24G08 Gal4 line allows the expression of the mGFP reporter gene specifically in the MNs **(A)** innervating the VAMs and was named VAMN Gal4 line (Ventral Abdominal Motor Neurons). One abdominal segment (A3) is presented here with HRP staining **(A')** used to label the nervous system. Merged images with HRP **(A”)** and phalloidin labeling the muscles **(A”')** are shown. **(B–B”)** The 31B08 Gal4 line is expressed specifically in the adult muscle precursors of the VAMs during the third instar larval stage. At low magnification (10X objective), two groups of GFP positive cells are visible per hemi-segment (A3-A4) on each side of the midline (white dashed line in **B)**. Larval muscles **(B')** and merged images are shown **(B”)** in a flat larva preparation. **(C-C””)** A higher magnification focusing on one group of GFP positive cells **(C)**. These cells express in their nuclei the transcription factor Zfh1 **(C',C”)**, an adult muscle precursor marker. Nuclei are Dapi stained (**C”',C””**). Muscles are labeled using phalloidin (in magenta). This Gal4 line was named VAMPC (Ventral Abdominal Muscles Precursor Cells). In all presented images the anterior side is up**. (D–E”)** Apoptosis induced by the expression of *reaper* with the VAMPC Gal4 line (UAS Reaper/+; VAMPC Gal4/+) results in adult flies lacking the majority of the abdominal ventral muscles (**D**, white asterisks). Only few ventral muscles could subsist (in A1 and A2 segments) compared to control **(E)**. HRP (in red) stains the nervous system (**D',E'**). The white boxes in **D** and **E** focus on A4 abdominal segment at higher magnification where the ventral muscles are absent in **D”** compared to the control condition in **E”**. Note that in the genetic condition UAS Reaper/+; VAMPC Gal4/+, the lateral muscles are not affected as shown by the phalloidin staining used to label muscles (in magenta). Abdomen is oriented anterior up and posterior down.

Our aim was to study specifically the VAMs to pinpoint their function in adult fly. In the search for a behavior associated to the function of the VAMs, we needed specific genetic tools to allow the manipulation of the corresponding motor neurons (the Ventral Abdominal Motor Neurons, VAMNs) as well as tool for the VAMs themselves. Recently, thousands of transgenic Gal4 lines have been generated and are known as the Janelia FlyLight stocks. In this collection, the Gal4 open reading frame is under the control of different short intronic (or noncoding) regions of genes known to have a patterned expression in the adult nervous system (Pfeiffer et al., 2008; Jenett et al., 2012). For each line the expression pattern is described and available on Janelia FlyLight database (http://flweb.janelia.org/cgi-bin/flew.cgi). Using this database, we first selected the enhancer Gal4 stocks that drive the expression of the GFP reporter in a subset of cells located in the abdominal ganglia, assuming that the cell bodies of the VAMNs are positioned in the abdominal ganglia of the ventral nerve cord (VNC). We eliminated all the lines showing too broad GFP expression pattern in the brain or in the entire VNC. We then screened the selected lines by crossing them with a UAS-GFP construct to study their expression pattern in MNs and NMJs of the VAMNs in adult. One line, 24G08 Gal4 showed a spatially restricted and specific expression pattern in the glutamatergic NMJs on the VAMs (Figures 2A–A”') in all the abdominal segments (data not shown). This 24G08 Gal4 line is specific of the VAMs since no GFP expression was detectable in the NMJs from the other abdominal motor neurons innervating the dorsal or the lateral muscles (Supplementary Figure 1). We called this specific tool: VAMN Gal4 for Ventral Abdominal Motor Neurons Gal4 line. Next, the VAMN Gal4 was tested for its expression pattern during the third instar larval stage. Notably, in larvae no GFP expression was found either in MNs or at the NMJs level, and only a subset of cells is labeled in the VNC (Supplementary Figure 2). This finding emphasized the importance of the VAMN Gal4 tool since this Gal4 line allows the expression specifically at the adult stage in specific motor neurons innervating the VAMs. Independently, we selected another Gal4 line for its specific expression during the larval stage in the precursor cells of the adult VAMs. The adult muscle stem cells called adult muscle precursors (AMPs) arise during the embryogenesis and stay undifferentiated throughout embryonic and larval stages (Bate et al., 1991; Currie and Bate, 1991; Figeac et al., 2010). AMPs are maintained quiescent due to the expression of Notch and Twist that target repressors of differentiation such as the *zinc finger homeodomain 1* (*zfh1*) and *Holes in muscle* (*Him*) genes (Anant et al., 1998; Bernard et al., 2010). When the expression of *twist* is downregulated, these cells proliferate and give rise to the adult muscles. We found that the 31B08 Gal4 line allows the expression of the GFP reporter specifically in a subset of small cells ventrally located and closely linked to the neighboring muscles on each side of the midline in larvae (Figures 2B–B”). We showed that these cells express the transcription factor Zfh1 (Postigo et al., 1999) known to be an AMPs marker (Figures 2C–C””) that most likely impedes the myogenic differentiation via Mef2 (Figeac et al., 2010). From their location and their Zfh1 expression, we thought that these cells are likely the AMPs for the abdominal ventral muscles. To further test this, we used the same Gal4 line and the pro-apoptotic cell death gene *reaper* to induce cell death. The resulting adult flies display a loss of the majority of the VAMs (Figures 2D–D”) compared to control flies (Figures 2E–E”). This phenotype is not completely penetrant and some VAMs are not affected and are formed normally. For example in Figure 2D, in abdominal segments A1 and A2 the VAMs are visible and in some cases, presence of VAMs could be observed in other segments as well. Nevertheless, this phenotype is specific because no other muscle in the abdomen is affected; the dorsal and lateral muscles are still present and unchanged (Figures 2D–D”). This result confirms that the 31B08 Gal4 line is expressed in a subset of the AMPs of the VAMs during the larval stage. We decided to call the 31B08 Gal4 line, VAMPC Gal4 for Ventral Abdominal Muscle Precursor Cells. Together these observations reveal that the VAMN and VAMPC Gal4 lines are two new genetic tools that will be very useful for the study of the VAMs function in the adult *Drosophila*.

### Monitoring the abdomen movements with a new behavioral test in adult: the folding of the abdomen

**Figure 3:**
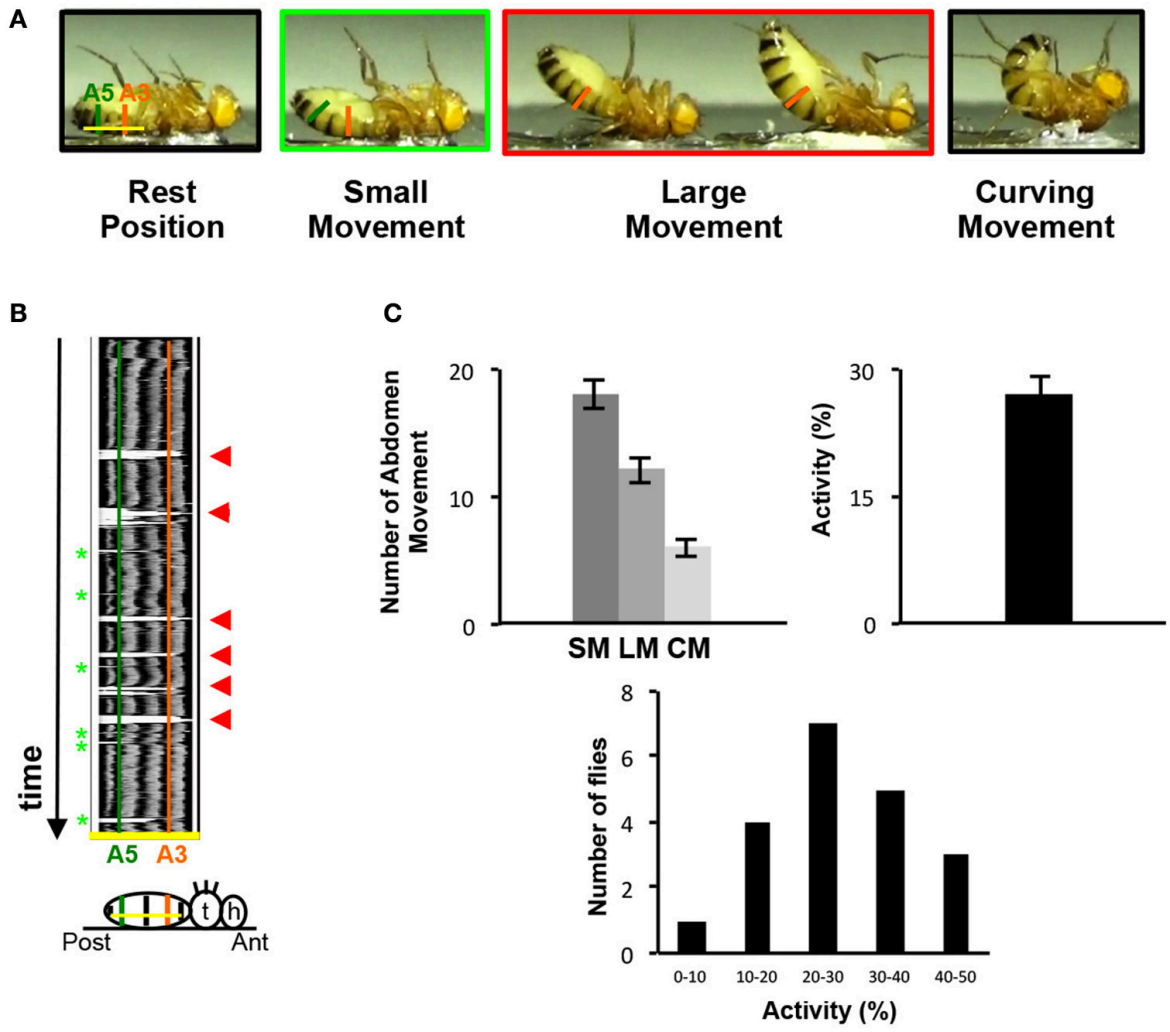
The abdomen folding: A new behavioral test. **(A)** Control adult flies, thorax fixed on a glass slice move their abdomen. Depending on the amplitude of the abdominal movements, small or large movements are distinguished. Taken from movie sequences, fly into light the green rectangle illustrates small movement (SM) and large movement (LM) is highlighted in the red rectangle. The two extreme positions (black rectangles) are the resting position when the abdomen is parallel to the glass slice (left) or at the opposite the total curving of the abdomen when the abdomen is perpendicular to the slice glass (right, curving movement, CM). The baseline is represented by a yellow line at the resting position and the segment A3 and A5 are showed by vertical line orange and green, respectively. **(B)** Video recording could monitor the abdomen folding movement. Kymograph plot from video recording of the abdominal folding movement, which presents the spatial position of the fly abdomen over time along an axis. This axis is determined for each fly at resting position by a line from A2–A6 (baseline, yellow line in 3A). Each abdominal segment shows a dark pigmented tergite part easily visible on the kymograph. A3 and A5 are represented by vertical lines orange and green respectively. The movements of the abdomen during the record are visualized by the presence of white peak zones that correspond to the absence of pigmented tergite at that time and position. Small movements are associated with small white peaks at A5 position (light green asterisks) and the large movements correspond to the absence of tergite from A6 until A3 and generate large white peaks (red arrowheads). Below the kymograph, a fly pasted on the back is drawn in resting position. At the right side the head (h), the thorax (t) and the abdomen are represented. On the abdomen the baseline is in yellow and a green and orange vertical line respectively show the A5 and A3 segments aligned with the same region in the upper kymograph. **(C)** General folding behavior parameters. The number of small and large movements is counted from kymographs. The number of curving movements is determined from the movie. 20 control flies have been recorded during 1 min twice, these flies perform 18 SM (±1.09), 12 LM (±0.98) and 6 CM (±0.7) in average and show 27% (±2.46) of activity. The percentage of activity clusters all the abdominal movements executed by the sample of flies in average. This percentage is obtained from the kymograph (see Materials and Methods). The distribution of the percentage of activity for each control fly tested is represented by histograms. The percentage of activity varies between 8 and 47% with the class between 20 and 30% activity the most represented. The percentage of activity is calculated from the 40 videos recorded. The error bars represent the standard error of the mean (±SEM).

Because of their location on the ventral part of the abdomen, the VAMs could likely be involved in folding or curving movements of the abdomen. We therefore designed a behavioral test to observe and monitor these movements. Since a fly placed on the back naturally lifts its abdomen to go back on its legs, we glued the back of adult flies on a microscope slide and video-recorded their movements (Figure 3A and for an example of movie see Supplementary Movie 1). The flies pull up their abdomen either slightly (up to A5 segment included) or in a more prominent position (up to A3 segment included). We named these movements small and large respectively (SM and LM). The most extreme position reached by the abdomen is the curving movement (CM). In this case, the tip (or the extremity) of the abdomen reaches a position perpendicular to the resting position (the horizontal position, parallel to the microscope slide) (Figure 3A). Thus, the abdomen of a fly is able to perform different folding movements detectable on movie analyses. We quantified these movements in two ways: (1) the number of occurrence of each type of movement (small, large, curving) during a 1-min period and (2) the relative percentage of time when the abdomen is not in a resting position during the 1-min video recording (the percentage of activity). To perform this, we used the Fiji software and analyzed each individual fly movement by measuring the displacement over time of the abdomen relatively to a drawn basal line corresponding to the resting position (yellow line in Figure 3B). On the representative kymograph (i.e., pixel intensity along the basal line over time), we can observe, when the abdomen stays in a resting position, the pigmentation pattern of the abdomen. When the abdomen folds, its position deviates from the basal line and a peak with background intensity (light gray) appears. The more the abdomen folds, the bigger is the corresponding peak in the kymograph. We tested a sample of 20 control flies in this behavioral test. The results reveal that a fly performs an average of 18 SM, 12 LM and 6 CM in 1 min, with 27% of activity on average (Figure 3C). Detailed analysis of these results shows that the number of abdomen movements for each fly varied between 8 and 28 for the SM, 3 and 17 for the LM and 1 and 14 for the CM (Supplementary Figure 3A). Control flies tested independently on different days for their abdomen movements show no significantly different results in the number of movements or in activity percentage (Supplementary Figure 3B). Finally, as a proof of principle, we have blindly analyzed twice the videos recorded for 20 control flies tested and the results obtained in the 2 independent analyses are not statistically different (Supplementary Figure 4). This indicates that our approach provides a robust and efficient method for analyzing the movements of the abdomen. Taken together, our data describe the design of a new behavioral test specifically adapted to the study of the abdomen movements in the adult fly.

### The VAMs are required for the folding movement of the adult fly abdomen

**Figure 4:**
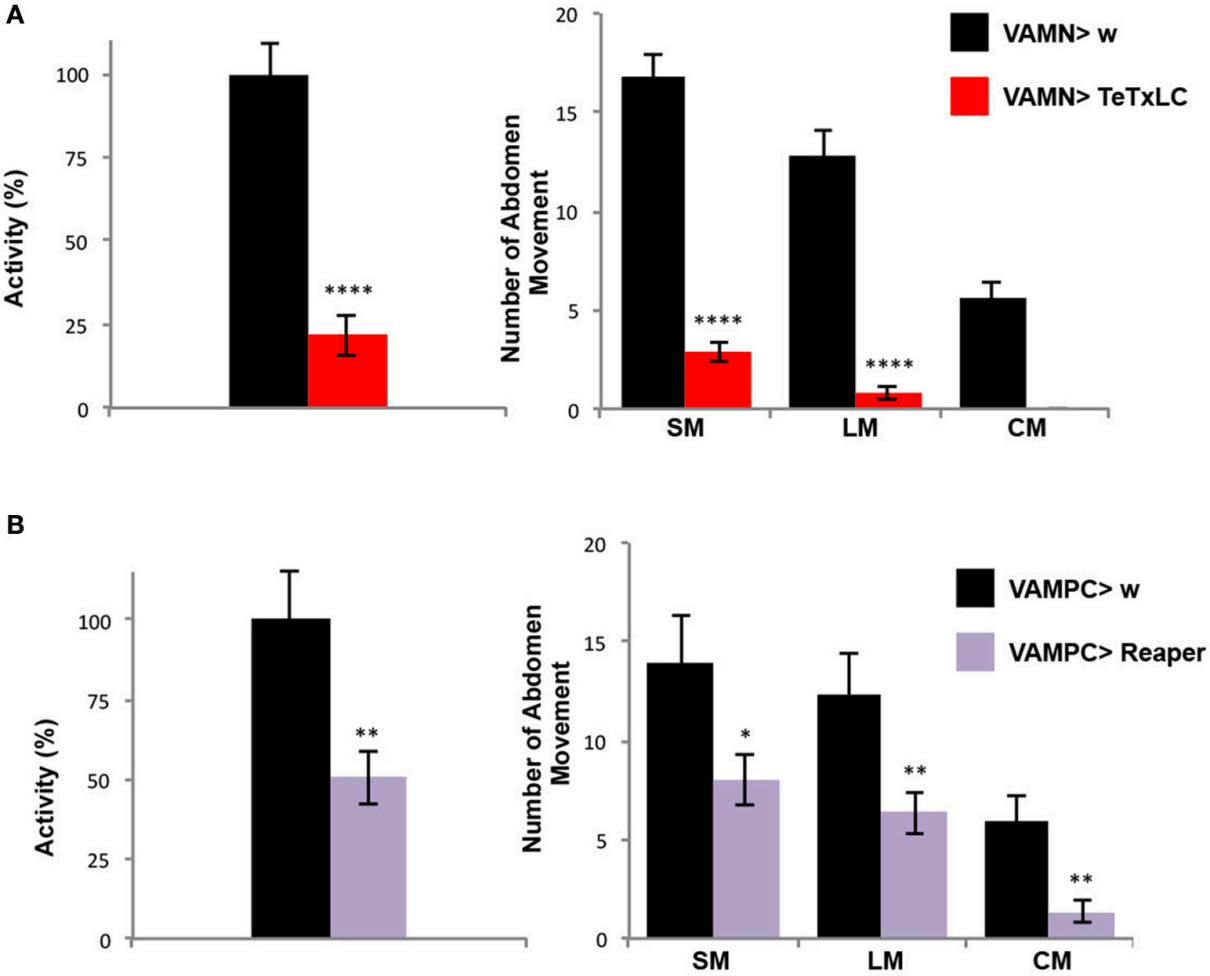
The VAMs are required for the abdomen folding behavior. **(A)** The specific inactivation of the synaptic transmission at the VAMs level using the expression of the tetanus toxin light chain (TeTxLC) leads to a drop of the abdomen folding activity. A reduction of 78% in activity of the abdomen movements is observed in VAMN Gal4/+; UAS TeTxLC/+ flies (in red, *n* = 31) compared to control flies, VAMN Gal4/+ (in black, *n* = 29). All types of movements (SM and LM) are affected and the abdomen curving (CM) is abolished when the synaptic transmission to the VAMs is impaired. Pool data are represented as means ± SEM, ^****^p < 0.0001. **(B)** Flies expressing *reaper* in the AMPs of the abdominal muscles (UAS Reaper/+; VAMPC Gal4/+) show a reduction by half of the folding abdomen activity (in purple, *n* = 29) compared to the control flies (VAMPC Gal4/+, in black, *n* = 15). This result is the consequence of an overall abdomen movement reduction. VAMPC>Reaper flies perform 8 SM, 6 LM and 1 CM compared to 14, 12 and 6 respectively in control flies. Pool data are represented as means ± SEM, ^*^p < 0.05, ^**^p < 0.01.

Once the folding behavioral test was set-up with control flies, we decided to use a genetic approach to address the question of the contribution of the VAMs during the folding movement of the adult abdomen. First we expressed the tetanus toxin light chain (TeTxLC) (Sweeney et al., 1995) under the control of the VAMN Gal4 line. The TeTxLC is known to cleave the Synaptobrevin, a vesicle-associated protein, and to inhibit specifically the release of neurotransmitters (Verderio et al., 1999). When the neurotransmission is impaired with the VAMNs Gal4 line, we observed a significant 78% decrease of the global abdomen activity compared to the control flies (Figure 4A). All the abdomen movements quantified (large and small movements) showed an important significant decrease in the frequency and the curving movement totally disappeared. These results show that the folding of the abdomen is disrupted when the neurotransmission is impaired in the MNs innervating the VAMs. To confirm these results we decided to address the question from the muscles' point of view. Hence, we used the VAMPC Gal4 line to express the pro-apoptotic gene *reaper* in the precursor cells of the adult VAMs at the larval stage (see Figure 2B). In that genetic context, the resulting phenotype in adult is the loss of the majority of the VAMs (see Figure 2D). These flies subjected to the folding abdomen behavior test display a 50% decrease of the abdomen activity compared to control flies. Although the loss of VAMs is incomplete, the abdominal activity and the number of abdominal movements performed by these flies are significantly decreased. All the movements of the abdomen are affected. The number of SM, LM, and CM are significantly reduced compared to control flies (Figure 4B). Our results show that the impairment of the neurotransmission at the NMJs level as well as at the loss of the VAMs abolish or reduce the folding movements of the abdomen.

Altogether, our results using different specific Gal4 strains, allow us to conclude that the VAMs are responsible for the folding movement of the abdomen that can be analyzed and quantified by the behavioral test we set up. This new behavioral test of abdomen folding is a read-out that correlates with the VAMs activation.

## Discussion

### The VAMs of the abdomen and their innervations in the adult fly

We chose to address the question of the role of the VAMs in the adult fly because these muscles are the largest of the abdomen. The VAMs are well grouped, on both sides of the ventral midline and the muscle fibers are organized parallel to this midline in each abdominal segment. The pattern of the VAMs is also stereotyped in the abdomen, with the segment A1 containing more laterally on both sides of the midline two other groups of muscles besides the muscular fibers at the midline. The A2 segment displays muscles in chevron pattern, easily distinctive. In this study, we did not image the muscles of these two segments because the segment A1 is cut at the dissection and in A2 two clusters of chordotonal organs brightly stained by HRP are located nearby muscles, making difficult the imaging of A2 muscle innervation. So the VAMs images we showed are representative of the abdominal segments A3–A5.

To study the VAMs activation and their movements, we looked at the innervations of these muscles. As reported previously by different studies, the VAMs receive a glutamatergic innervation but the precise number of these MNs is not known. The glutamatergic NMJs present different sizes of boutons with an ultrastructure similar to what has been described in L3 larvae (Hebbar et al., 2006; Wagner et al., 2015). In our VAMs preparations, we also observed the presence of thin lines of very small varicosities closely aligned to glutamatergic boutons that we have identified as octopaminergic/tyraminergic innervation. In adult, this type of innervation was already described in the prothoracic muscles (Rivlin et al., 2004). As in larvae, the octopamine/tyramine innervation is very labile from a fly to another. Indeed, the number of octopamine/tyramine varicosities is variable and can be absent in some flies (data not shown). Octopamine, in larvae, is known as a regulator of synaptic functions for adaptations to environmental changes, for instance in response to starvation conditions (Koon et al., 2011). In adult, the octopamine has been reported to modulate aggression, egg laying behavior or sleep (Monastirioti et al., 1996; Baier et al., 2002; Crocker et al., 2010). So far, the role of octopaminergic innervation of the VAMs has not been investigated and further studies will be required to understand the action of octopamine on VAMs contractions.

### A new behavioral test in adult

Because of the VAMs position along the vertical ventral midline, we assumed that these muscles could be involved in the bending movement of the abdomen toward the thorax. Such a situation occurs when a fly put on its back tries to land on its legs in a survival escape reflex. For that purpose, we designed a behavioral test to monitor and quantify the folding movement of the abdomen. Interestingly, in the two genetic conditions that led either to the inactivation of the synaptic transmission or to the loss of the majority of the VAMs tested, the folding movement of the abdomen was significantly reduced but not abolished. This suggests that other abdominal muscles could be engaged in this folding movement. Earlier descriptive studies classified the lateral abdominal muscles as compressors and the dorsal muscles to tergites retractors (Miller, 1950). The specific function of these muscles has not been studied yet, but we propose now that with the VAMs they help in the refinement of the abdomen-folding movement occurring when a fly is on its back.

Others adult behaviors like the mating behavior in male or egg deposition in female also engage somehow the action of the abdomen muscles and likely the VAMs themselves are involved in. Nevertheless, the abdomen folding movement that we have described and quantified has the major advantage to allow monitoring the specific activation of only one class of muscles, the VAMs. Our study establishes a link between some specific motor neurons and a given related behavior, the folding of the abdomen. This is a first step for further investigations to understand the neural network underlying this precise behavior with the identification of second order ascending interneurons. On the other hand, the folding behavior of the adult abdomen could be used in studies on muscle degeneration. The folding behavior also follows the ability of the VAMs to contract and as a result could thus also be employed in muscle degeneration paradigms.

### Genetic new tools: the flylight Gal4 collection

The Enhancer Gal4 lines of the Janelia Light Project provide to the community a huge collection of Gal4 lines. This collection offers several interesting particularities. The small size of the enhancer-DNA fragments cloned upstream of the Gal4 gene result in restricted expression pattern, allowing ectopic gene expression specifically small tissue regions or within a small number of cells. In addition, the full expression pattern (in embryo, larva and adult) of these different Gal4 lines is documented and available on line. We have selected from the database, accordingly to their expression profile in the abdominal ganglia, nearly 200 enhancer Gal4 lines. These lines have been screened for their expression pattern in the MNs of the VAMs. Only one line, 24G08 Gal4 allows the expression of the GFP reporter specifically in the MNs innervating the VAMs of the abdomen. Using this Gal4 line, neither the MNs innervating the lateral nor the dorsal muscles of the abdomen are targeted. In addition, this strain is expressed in the MNs of the VAMs only at the adult stage and not during the larval development. This is a huge advantage compared to the widely used VGlut Gal4 or D42 Gal4 lines expressed in all MNs since the embryonic stage.

It is interesting to note that the fragment 24G08 (VAMN Gal4) is an enhancer of the *diuretic hormone receptor* (DH31-R) gene. This receptor belongs to the class II G protein-coupled receptor (GPCR) gene family and DH31-R is the calcitonin gene-related receptor vertebrate homologous (Johnson et al., 2005; Cardoso et al., 2014). DH31-R is expressed in corazonin neurons that are peptidergic interneurons located in the brain and in the VNC (Johnson et al., 2005). The VAMN Gal4 seems to be at least a sub-pattern of DH31-R pattern itself. A role of DH31-R into the MNs controlling the folding movements of the abdomen could be imagined. The signaling pathways of this GPCR as well as its targets are still unknown and will require further studies. The fragment 31B08, that drives the Gal4 expression in the AMPs of the VAMs during the larval stage (VAMPC Gal4), belongs to the gene *slit*. Slit is well known to be expressed by tendon cells and to exert a function in the muscle migration process (Kramer et al., 2001; Wayburn and Volk, 2009; Ordan et al., 2015). In this way, its expression into the AMPs is difficult to decipher. It also needs to be borne in mind that the enhancer activities of the Janelia Gal4 lines deprived of all their natural DNA background (other enhancers, silencers) might not reflect the actual expression pattern of the genes. These expression patterns have to be confirmed by RNA or protein detection before starting new studies for potential roles of DH31-R or Slit in MNs or muscles development.

During metamorphosis, great changes occur and both the muscles and their innervation have to be rebuilt. Soon before eclosion at 90H APF (after pupa formation) the adult neuromuscular system is already built (Hebbar et al., 2006), with the MNs innervating their target muscles. Nevertheless, the neuromuscular system of the newly born adult is reshaped during the first 5 days after eclosion (Rivlin et al., 2004). Indeed, during that time, the length of synaptic branches, the size of the boutons and the number of active zones change (our unpublished observations). These modifications are the last developmental steps of the neuromuscular junctions, after which these synapses will be maintained during the adulthood.

Further studies are necessary to answer remaining questions. How the adult neuromuscular system is stabilized in young adult? What are the signaling pathways involved during this synapse remodeling? We will investigate these questions using the genetic tools we described to target either the pre- or the post- synapse specifically in the adult with the abdomen folding behavioral test as a muscle activity read out.

## Ethics statement

*Drosophila melanogaster* flies as invertebrate animals are exempted from ethics approval.

## Author contributions

MP and CS performed experiments and analyzed data. LS provided technical advice and contributed intellectually. M-LP conceived Fiji analyses of movies and contributed intellectually. YG conceived behavioral test experiments and analyzed data. SL conceived the project, designed and performed experiments, analyzed data and wrote the manuscript with M-LP, YG, and LS contributions.

### Conflict of interest statement

The authors declare that the research was conducted in the absence of any commercial or financial relationships that could be construed as a potential conflict of interest.

## Abbreviations

AMPs: Adult Muscle Precursors
CM: Curving Movement
DFMs: Direct Flight Muscles
HRP: Horse Radish Peroxidase
IFMs: Indirect Flight Muscles
LM: Large Movement
mGFP: Membrane Green Fluorescent Protein
MNs: Motor Neurons
NMJs: NeuroMuscular Junctions
SM: Small Movement
SSR: Subsynaptic Reticulum
VAMs: Ventral Abdominal Muscles
VAMNs: Ventral Abdominal Motor Neurons
VAMN Gal4: Ventral Abdominal Motor Neurons Gal4
VAMPC Gal4: Ventral Abdominal Muscle Precursor Cells Gal4
VNC: Ventral Nerve Cord.
