# Supplementary material for: A New Behavioral Test and Associated Genetic Tools Highlight the Function of Ventral Abdominal Muscles in Adult Drosophila
Source: Front Cell Neurosci. 2017 Nov 21;11:371. doi: 10.3389/fncel.2017.00371 (PMC5702315; doi:10.3389/fncel.2017.00371)
Supplement: Supplementary file 2 [file DataSheet1.DOCX]

Supplementary Material

**A new behavioural test and associated genetic tools highlight the function of ventral abdominal muscles in adult *Drosophila*.**

**Marine Pons^1,2^, Claire Soulard^1,3^, Laurent Soustelle^1^, Marie-Laure Parmentier^1^, Yves Grau^1^, Sophie Layalle^1,*^**

1 IGF, CNRS, INSERM, Univ. Montpellier, F-34094 Montpellier, France

2 present address: Normandie Univ, UNIROUEN, INSERM U1245, IRIB, Rouen, France

3 present address: The Institute for Neurosciences of Montpellier, Inserm UMR1051, Saint Eloi Hospital, Montpellier, France; Université Montpellier, France.

*** Correspondence:**

Sophie Layalle

sophie.layalle@igf.cnrs.fr

**Running Title:** New behavioral test for the *Drosophila* ventral abdominal muscles.

**Supplementary Figures**

**Supplementary Figure 1**: **24G08 Gal4 is specifically expressed in the MNs innervating the ventral abdominal muscles.**

Motor neurons innervating the dorsal, lateral and ventral muscles of the abdomen (A, B and C respectively) are stained by HRP (in magenta) in 24G08 Gal4>mGFP adult flies. No GFP is observed in the dorsal (A’) and lateral (B’) motor neurons or NMJs. The GFP is found expressed in the motor neurons and NMJs innervating the ventral muscles of the abdomen (C’). Merge images for HRP and GFP are presented (A’’, B’’ and C’’). In 24G08 Gal4>mGFP flies co-staining is only observed in the MNs innervating the ventral muscles (C’’).

**Supplementary Figure 2**: **VAMN Gal4 is not expressed at the larval NMJ.**

(A) The VAMN Gal4 line does not drive the expression of the GFP in the MNs and NMJs in third instar larvae. Lower panels are zoomed images of the region outlined in white in the upper left picture. No GFP staining is visible at the synaptic boutons in larvae.

(B) In the larval VNC, the VAMN Gal4 line conducts the GFP expression in a subset of cells but no expression is found in motor neurons. These cells seem to be corazonin neurons that are peptidergic interneurons (see Discussion). Note that VAMN Gal4 is also likely to be expressed in some additional interneurons.

The nervous system is stained by HRP in red, the GFP is in green and the muscles are labeled by phalloidin in magenta.

**Supplementary Movie 1: An example of the abdomen folding movement.**

**Supplementary Figure 3**: **Abdomen folding behavioral test: details and reproducibility.**

(A) The different histograms show the distribution of the number of small (1), large (2) and curving (3) movements of the 20 control flies individually. 1: The number of SM varies between 8 and 28, with an average of 18 SM. 2: The number of LM varies between 3 and 17 and the average is 12. 3: The number of CM is comprised between 1 and 14 with an average of 6. (The total of the control flies tested is 20.)

(B) Three cohorts of control flies tested independently for the abdomen folding behavior show no significant difference in the parameters analyzed. The number of LM varies from 9 to 11, the number of SM varies from 11 to 16 and the activity percentage varies from 20 to 28%. These results are not statistically significant since all p-values (calculated by t-tests) are up to 0.05. Cohort 1 (n=11), Cohort 2 (n=8), Cohort 3 (n=14).

**Supplementary Figure 4**: **Abdomen folding behavioral test: analysis of the videos recorded.**

A group of 20 control flies was subjected to the abdomen folding behavioral test and videos recorded one minute twice. For each fly, the videos have been blind tested two times on separate days and the number of SM, LM, CM and the activity percentage calculated. The results obtained from the two independent analyses (Analysis 1 and 2) are not statistically different. T test (Tt) calculated between the analysis 1 and 2 for the parameters tested: SM, Tt=0.28; LM, Tt=0.84; CM, Tt=0.97; Activity, Tt=0.3.
