# Supplementary figures and images for: A New Behavioral Test and Associated Genetic Tools Highlight the Function of Ventral Abdominal Muscles in Adult Drosophila
Source: Front Cell Neurosci. 2017 Nov 21;11:371. doi: 10.3389/fncel.2017.00371 (PMC5702315; doi:10.3389/fncel.2017.00371)

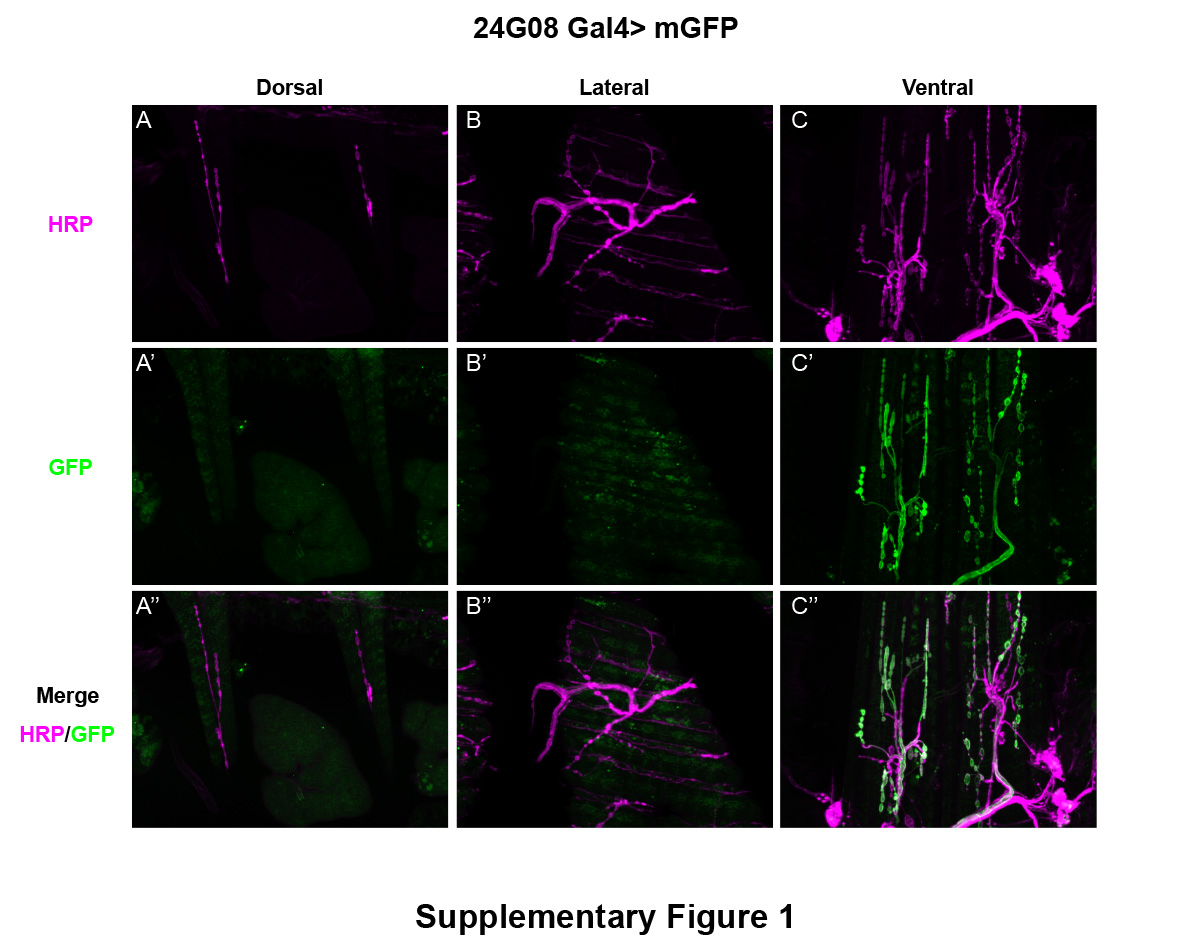

Supplement: Supplementary file 3 [file Image1.jpg]

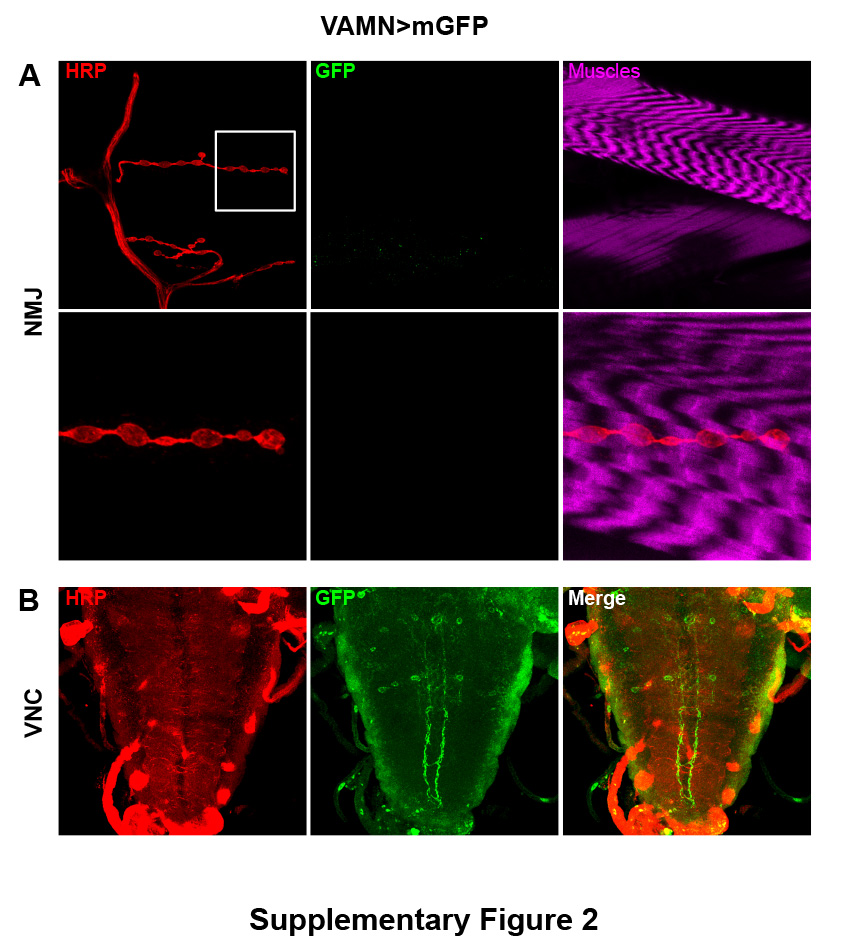

Supplement: Supplementary file 4 [file Image2.jpg]

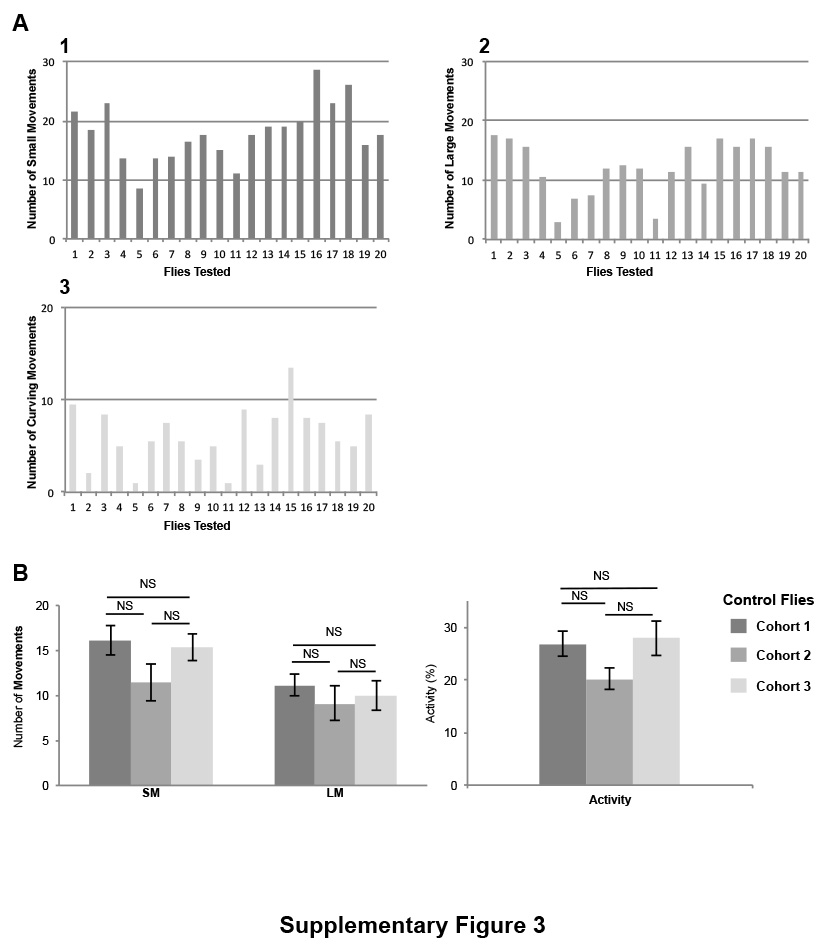

Supplement: Supplementary file 5 [file Image3.jpg]

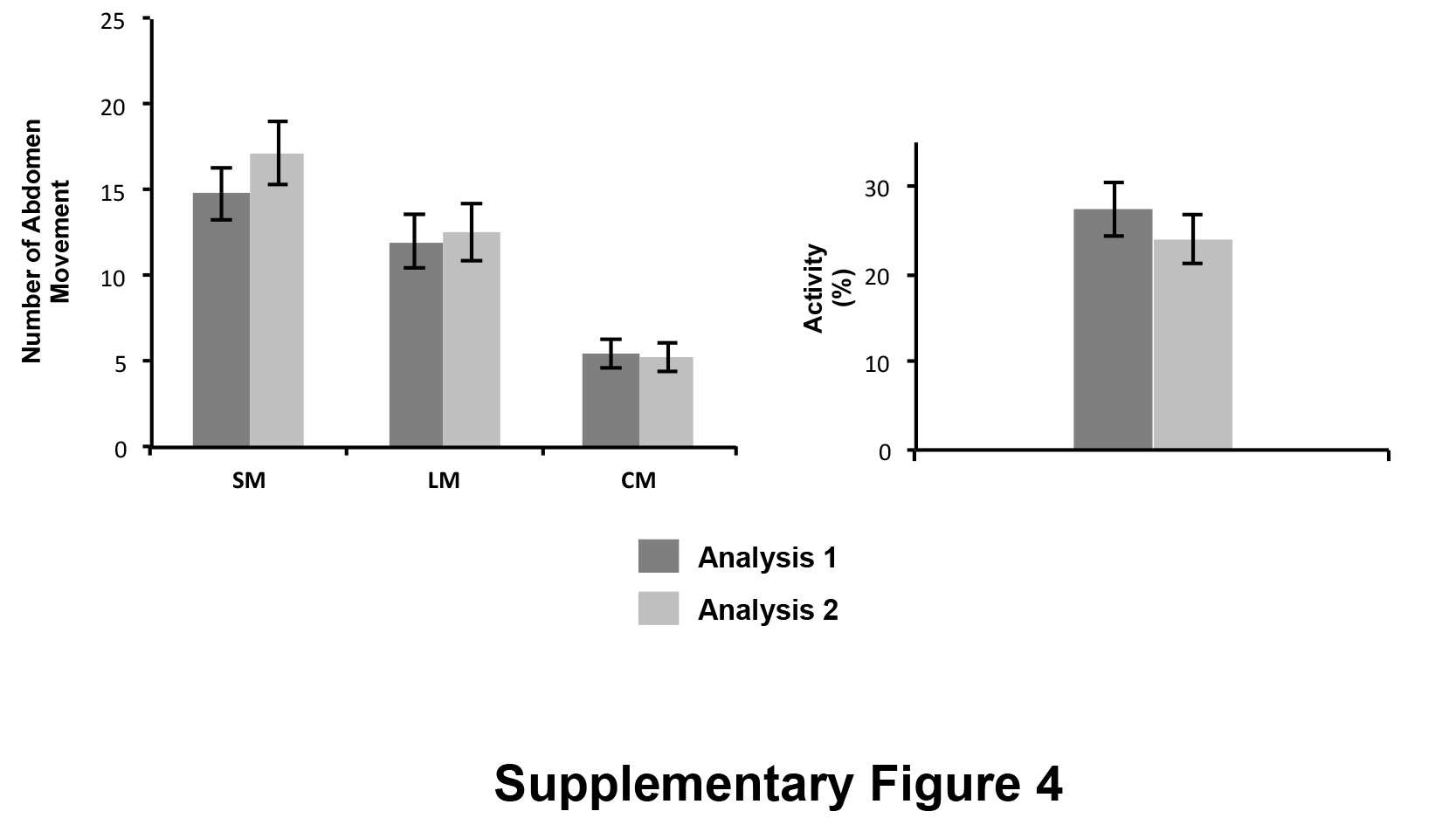

Supplement: Supplementary file 6 [file Image4.jpg]
